# Design of vortex-based cavitation devices/reactors: Influence of aspect ratio, number of inlets and shape

**DOI:** 10.1016/j.ultsonch.2023.106695

**Published:** 2023-11-22

**Authors:** Amol Gode, Ketan Madane, Vivek V. Ranade

**Affiliations:** Multiphase Reactors and Intensification Group (mRING), Bernal Institute, University of Limerick, Ireland

**Keywords:** Hydrodynamic cavitation, Swirl flow, Pressure drop, CFD, Emulsion

## Abstract

Vortex-based hydrodynamic cavitation devices are being used in a wide range of applications. However, adequate information on the design of such devices is not available. In this work, we have computationally investigated the influence of key design parameters such as the aspect ratio of the vortex chamber, the number of tangential inlets and the shape of the device on resulting flow characteristics and cavitation. Experiments were carried out to validate key findings from the computational studies. These investigations revealed that the aspect ratio of the vortex chamber as six may be considered as optimum. The performance of single and multiple inlet devices was found to be comparable at the same pressure drop (that is at same energy consumption per m^3^). Scale-up with a geometric similarity led to a reduction in the extent of cavitation for same energy consumption per m^3^. For facilitating scale-out option, an attempt was made to simplify the configuration of the vortex-based cavitation device. Computational results indicated that the cavitation performance of simplified configuration was not significantly inferior. A case of the formation of liquid–liquid emulsion was taken as a test case for evaluation of a modified cavitation device based on the present investigations. The droplet size distributions of emulsions generated by both the devices indicate that the proposed simplified configuration, which may facilitate fabrication and offer integrated scale-out options, performs almost at par with a complex configuration. The presented results will be useful for optimising designs of vortex-based hydrodynamic cavitation devices/ reactors.

## Highlights

• Evaluated influence of key design parameters of vortex based hydrodynamic cavitation devices.
• The aspect ratio of the vortex chamber as six was found to be optimum.
• Multiple units of single inlet device perform better than single unit of multiple inlet device.
• Appropriate simplifications in the device geometry are developed without compromising the performance.

## Nomenclature

D: chamber diameter, mm
$d_{T}$: throat diameter, mm
Eu: Euler number, -
$V_{T}$: throat velocity, m/s
$V_{\theta }$: tangential velocity, m/s
$V_{\mathrm{vap}}$: volume of vapour, m^3^
$V_{D}$: total volume of device, m^3^
ρ: density, kg/m^3^
μ: viscosity, Pa.s
Ret: throat Reynolds number, -
*n_I_*: number of inlets, -
τ: residence time, s
*Q*: flow rate, m^3^/s
*V*: Volume, m^3^
*t*: time, s
$n_{p}$: number of passes, -
Γ: circulation, m2/s
r: radial distance, m
$r_{c}$: critical radial distance, m
$V_{\theta max}$: maximum tangential velocity, m/s
$P_{\min}$: minimum pressure, Pa
$P_{\infty }$: maximum pressure, Pa
$r_{\max}$: maximum radial distance, m
$P_{\mathrm{device}}$: power consumption of device, W
$d_{i}$: droplet diameter in i^th^ bin, μm
$d_{32}$: Sauter mean diameter, μm
$d_{320}$: Sauter mean diameter of pre-emulsion, μm
$N_{B}$: Number of bins, -
η: drop breakage efficiency, %
$E_{m}$: energy consumption per kg of product, W/kg
ΔP: pressure drop across the device, kPa
$\alpha_{0}$: volume fraction of oil, -
σ: interfacial tension, N/m

**Acronyms**
*CFD*: Computational Fluid Dynamics
*HC*: Hydrodynamic Cavitation
*VD*: Vortex-based cavitation devices
*PRESTO!*: PRESsure Staggered Option
*SIMPLE*: Semi-Implicit Method for Pressure-Linked Equations
*URANS*: Unsteady Reynolds Averaged Navier-Stokes
*SST*: Shear Stress Transport
*BOI*: Body of Influence

## 1 Introduction

Vortex-based hydrodynamic cavitation devices (VD) are finding increasing applications in a wide range of industries [1]. Most of the information in the published studies is based on the designs disclosed in the patents [2], [3]. These patented designs disclose the vortex diode as a cavitation device without any moving parts. These designs consist of at least one tangential inlet port connected to a vortex chamber and having an axial outlet port. Previously, the operation of the vortex diode has been studied both in the forward and reverse direction. The forward direction being the one where the inlet is from the axial port and the reverse direction has inlet from the tangential port. It has been observed that higher resistance occurs in reverse flow as compared to forward flow operation, which resulted in these devices being used as non-return valve with small leakage. In the present study, the use of vortex diode as a hydrodynamic cavitation device is investigated when flow through the device is in the reverse direction i.e., from the tangential inlet port to the chamber and out from the axial port.

Vortex device when operated in this mode, the pressure is high at the periphery of the chamber and drops in the centre of the chamber often approaching the vapor pressure depending on the tangential velocities generated. The low pressures at the centre of the chamber leads to formation of cavities under certain conditions. These cavities collapse when they experience turbulent pressure fluctuations while travelling to high-pressure regions. These collapsing cavities generate intense shear, local hot spots and oxidising radicals which are typically called as cavitation effects. These cavitation effects lead to physico-chemical changes in the flowing stream and are harnessed in various applications to produce desired transformations. Despite the widespread applications of the vortex-based cavitation device, most of the published studies use standard device configuration as disclosed in the patents barring an exception of Kulkarni et al., [4], [5]. The work of Kulkarni et al., was focussed on diodicity of the device and on non-cavitating regime. Most of the interesting applications of vortex-based device are based on cavitating flows [1], [6], [7], [8]. Despite numerous applications, adequate information on key design parameters of vortex-based cavitation devices is not available. In this work, we have computationally investigated the influence of key design parameters of vortex-based devices/ reactors. Prior work on the design of such vortex-based devices, though primarily in non-cavitating regime, is briefly reviewed in the following.

The earliest efforts to understand the effect of geometrical design on the performance of vortex diode is published by Priestman [9], [10] and Vatistas et al., [11]. For incompressible non-cavitating flow, Priestman studied the effects of changes in geometry on performance. According to Priestman, the optimum aspect ratio ($D/d_{T}$, where D is the chamber diameter and $d_{T}$ is the throat diameter) was about 6 and varying this in the range 5 to 8 had only relatively small effect. Vatistas et al., through experiments and theoretical investigations, concluded that the core size and radial pressure distribution are functions only of the geometric parameters of the chamber. Along similar lines, the study by Kulkarni et al., [4], [5], providing design guidelines for vortex diodes based on diodicity; Pandare and Ranade [12] investigated through single-phase CFD simulations the vortex flow generated in vortex diodes; Simpson and Ranade [13] included the effect of scale on the performance of the vortex diode and Thaker and Ranade [7] studied the emulsion formation by a vortex diode of a particular configuration. The study involving impact of geometrical parameters on the performance of vortex diode is needed to understand and optimize the vortex diode design for applications harnessing cavitation.

Based on the works of Priestman [9], [10] and Kulkarni et al., [4], [5], it could be inferred that the inlet-to-outlet port diameter ratio should be one and the vortex chamber width equal to the diameter of the inlet/outlet port. The vortex diode design parameters suggested by Kulkarni et al., [4], [5], the entry and exit port size same as height of the chamber for higher diodicity, are maintained same in the present study. The other design parameters considered here include the aspect ratio which is the ratio of chamber diameter to throat diameter, number of inlets to the vortex diode and scale of the vortex diode defined in terms of throat diameter. The results from Kulkarni et al., (2008) indicates that for low Re, the aspect ratio value of ∼ 5 gives better performance as compared to slightly higher or lower aspect ratio. Moreover, they considered aspect ratio values lower than six based on the diodicity results of Priestman [10]. It is important to examine the influence of higher values of aspect ratio (more than 6). There are some studies reporting use of multiple inlets [14], [15], [16]. However, no systematic information on the influence of number of inlets on cavitating flow in vortex-based devices is available. Recently, Thaker and Ranade [7] and Ranade et al., [17] investigated the effect of scale on the performance of lab-scale and bench-scale vortex based cavitation device for the production of emulsions and wastewater treatment, respectively. It was observed that the performance of the cavitation device reduced with an increase in scale of the device. In this work, we computationally investigated influence of scale on flow and extent of cavitation. The focus was on understanding possible interaction of scale with aspect ratio and number of inlets. No such information is available. Based on the results of these investigations (discussed later), we investigated influence of simplified configuration of the device for facilitating potential scale-out possibility. The goal of simplifying the configuration was to facilitate ease of manufacturing and the possibility of using 3D printed devices for integrating multiple devices on a single platform [18].

Considering this critical review of the published literature, we have investigated the influence of the aspect ratio of the vortex chamber, the scale of the device, the number of inlets and simplified geometry of chamber and inlet/ outlet ports. Specific experiments were carried out to validate the computational models. The performance of final recommended configuration was compared with the standard configuration at the end. The approach and results presented in this work will be useful for developing improved vortex-based cavitation devices/ reactors.

## 2 Computational modelling

**Fig. 1:**
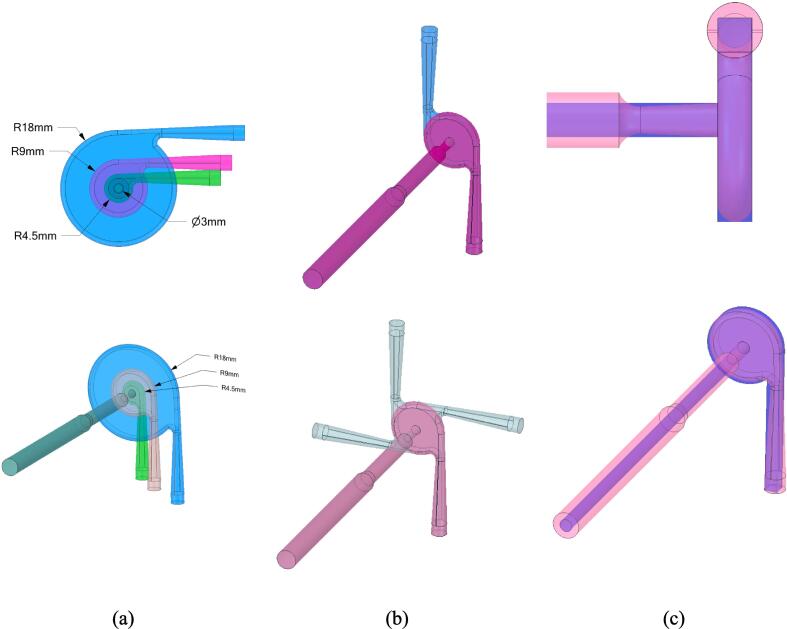
Configuration of vortex-based cavitation devices considered in this work (a) Aspect ratio, *D/dT* = 3, 6, 9, 12; (b) Number of inlets, 1, 2 and 4; (c) Shape: smooth and sharp contours.

Vortex-based cavitation devices (VD) generate highly vortical flow by virtue of their design. This highly vortical flow causes a low-pressure region at the core of the precessing vortex facilitating cavitation. It is essential to model the unsteady flow to understand the complex multiphase flow dynamics in the VD. The geometries of VD considered in this work are shown in Fig 1.

The computational model, boundary conditions and computational grid are discussed in the following.

### 2.1 Computational model

Water was used as a primary working fluid with a constant density (ρ) of 1000 kg/m^3^ and a constant dynamic viscosity (μ) of 0.001 kg/m-s (Pa.s). For the secondary phase, water vapour with a constant density (ρ) of 0.5542 kg/m^3^ and a constant dynamic viscosity (μ) of 1.34 x 10^-5^ kg/m-s (Pa.s) was used. Air (ρ = 1.225 kg/m^3^ and μ = 1.78 x10^-5^ kg/m-s) was also used for considering the dissolved air. For considered designs of VD, the throat Reynolds number (Ret=ρVTdTμ where ρ is the density of the fluid,$V_{T}$ is the average throat velocity, $d_{T}$ is the throat diameter, and μ is the dynamic viscosity) ranges from 7500 to 120000, indicating the turbulent regime of the flow. Selecting an appropriate turbulence closure and cavitation model to simulate the flow in the VD is essential. Different models are reported in the literature to simulate flow in vortex-based cavitation devices [7], [13]. The use of the Unsteady Reynolds averaged Navier Stokes (URANS) SST k-ω model to model and predict the unsteady flow dynamics in the vortex-based cavitation devices has been reported in the literature [7], [13]. Considering the qualitative and quantitative results obtained by the URANS SST k-ω turbulence model in different configurations of vortex-based cavitation devices reported in the literature, URANS SST k-ω was used in this work. The relevance of URANS SST k-ω turbulence model for simulating flow in vortex based HC devices is discussed by Simpson and Ranade [13] and is briefly summarised in Section S1 of supplementary information (SI). The multiphase flow was simulated for three phases, water (primary), water vapour (through cavitation) and air (dissolved air) with the Eulerian mixture model. The cavitation in the VD was simulated by the model proposed by Singhal et al., [19] and reported to have been used by Thaker and Ranade [7] and Simpson and Ranade [13]. For additional details of the computational model used in this work, the work of Simpson and Ranade [13] may be referred. For the sake of completeness, key aspects of the computational model and rationale behind them are briefly summarised in Section S2 of SI. The model equations are briefly listed here. The standard textbooks [20], [21], [22], [23], [24] and Ansys Fluent Theory Guide [25] may be referred for detailed discussion.

Continuity

(1) $$\frac{\partial \rho_{m}}{\partial t}+\nabla \cdot \rho_{m}{\vec{v}}_{m}=0$$

${\vec{v}}_{m}$(m/s) is the mass-averaged velocity and is defined as:

(2) $${\vec{v}}_{m}=\frac{\sum_{k=1}^{q}\alpha_{k}\rho_{k}{\vec{v}}_{k}}{\rho_{m}}$$

$\rho_{m}$(kg/m^3^) is mixture density given as:

(3) $$\rho_{m}=\sum_{k=1}^{q}\alpha_{k}\rho_{k}$$

$\alpha_{k}$(-) is the volume fraction of phase k, and q is the total number of phases

Momentum

(4) $$\frac{\partial \rho_{m}{\vec{v}}_{m}}{\partial t}+\nabla \cdot \rho_{m}{\vec{v}}_{m}{\vec{v}}_{m}=-\nabla P+\nabla \cdot \left[\mu_{m}\left(\nabla {\vec{v}}_{m}+{\vec{v}}_{m}^{T}\right)\right]+\rho_{m}g+F-\nabla \cdot \left[\sum_{k=1}^{q}\alpha_{k}\rho_{k}{\vec{v}}_{\mathrm{dr},k}{\vec{v}}_{\mathrm{dr},k}\right]$$

(5) $$\mu_{m}=\sum_{k=1}^{q}\alpha_{k}\mu_{k}$$

$\mu_{m}$(kg/m-s) the viscosity of mixture and F is the external body forces

(6) $${\vec{v}}_{\mathrm{kA}}={\vec{v}}_{k}-{\vec{v}}_{A}$$

The drift velocity ${\vec{v}}_{\mathrm{dr},k}$ (m/s) and slip velocity ${\vec{v}}_{\mathrm{kA}}$ (m/s) is related as

(7) $${\vec{v}}_{\mathrm{dr},k}={\vec{v}}_{\mathrm{kA}}-\sum_{k=1}^{q}c_{k}{\vec{v}}_{\mathrm{kA}}$$

${\vec{v}}_{\mathrm{dr},k}$ is the drift velocity (m/s), $c_{k}$ (-) mass fraction of the any phase given as:

(8) $$c_{k}=\frac{\alpha_{k}\rho_{k}}{\rho_{m}}$$

${\vec{v}}_{\mathrm{kA}}$ is slip velocity (m/s)

(9) $${\vec{v}}_{\mathrm{kA}}=\frac{24}{Re}\frac{\left(\rho_{k}-\rho_{m}\right)d_{k}^{2}}{18\mu_{A}C_{D}}\left(g-\frac{Dv_{m}}{\mathrm{Dt}}\right)$$

(10) CD=24Re1+0.15Re0.687Re≤10000.44Re>1000whereRe=ρmv→kAdkμA

Turbulence

(11) $$\frac{\partial }{\partial t}\left(\rho k\right)+\frac{\partial }{\partial x_{i}}\left(\rho ku_{i}\right)=\frac{\partial }{\partial x_{j}}\left(\Gamma_{k}\frac{\partial k}{\partial x_{j}}\right)+G_{k}-Y_{k}+S_{k},$$

(12) $$\frac{\partial }{\partial t}\left(\rho \omega \right)+\frac{\partial }{\partial x_{i}}\left(\rho \omega u_{i}\right)=\frac{\partial }{\partial x_{j}}\left(\Gamma_{\omega }\frac{\partial \omega }{\partial x_{j}}\right)+G_{\omega }-Y_{\omega }+S_{\omega },$$

k: Turbulence kinetic energy (m^2^/s^2^), G_k_ and G_ω_ are the generation of turbulence kinetic energy k and ω, $\Gamma_{k}$ and $\Gamma_{\omega }$ represent effective diffusivity of k and ω.

Cavitation

(13) $$\frac{\partial }{\partial t}\left(\rho_{m}f\right)+\nabla \cdot \left(\rho_{m}{\vec{v}}_{m}f\right)=\nabla \cdot \left(\Gamma \nabla f\right)+R_{e}-R_{c}$$

f is the vapor mass fraction (-),Γ effective diffusion coefficient, $R_{e}$ and $R_{c}$ are the mass source and sink terms for the evaporation and condensation, respectively formulated as:

(14) $$R_{e}=C_{1}\frac{\sqrt{k}}{\sigma }\rho_{v}\rho_{A}{\left[\frac{2}{3}\left(\frac{P_{v}-P}{\rho_{A}}\right)\right]}^{1/2}\left(1-f_{v}-f_{g}\right)$$

(15) $$R_{c}=C_{2}\frac{\sqrt{k}}{\sigma }\rho_{v}\rho_{A}{\left[\frac{2}{3}\left(\frac{P-P_{v}}{\rho_{A}}\right)\right]}^{1/2}f_{v}$$

$\rho_{v}$ is the density of the vapor (kg/m^3^), $f_{g}$ represents the mass fraction of non-condensable gases, $C_{1}$ and $C_{2}$ are empirical constants having value of 0.02 and 0.01, respectively [26].

### 2.2 Boundary condition and solution to model equations

The inlet surface of circular cross-section was considered as a velocity inlet. A flat velocity boundary condition of a constant velocity magnitude was used at the inlet surface. The direction of the velocity was kept normal to the inlet surface. The outlet surface of circular cross-section was defined as pressure outlet. The outlet section of the VD was extended for 50 d_T_ to avoid the reverse flow in the domain. The inlet velocity magnitude ($V_{\mathrm{in}}$) was calculated for all different scales and number of inlets to keep the outlet throat velocity ($V_{T}$) at 2.93 m/s. This was done to compare all the designs with different aspect ratios ($D/d_{T}$ - *D* is the diameter of the vortex chamber and $d_{T}$ – is the outlet throat diameter), number of inlets (*n_I_*) and scales (*S*) for same outlet throat velocity. All the simulations were carried out at least for a flow time of 3τ, where $\tau =\frac{V_{D}}{Q}$. Pressure drop, minimum volumetric pressure in the entire domain and the volume of the vapor generation were monitored and it was observed that a quasi-steady state was achieved after τ. After 1τ time-averaging of all the field variables was enabled to get the time-averaged values of the field variable for further post-processing and evaluation.

The model equations were numerically solved by the finite volume method [20], [21], [22], [23] using the commercial CFD code Ansys Fluent (Ansys Inc, Version 2022 R1 | https://www.ansys.com/). SIMPLE algorithm was used to couple the pressure and velocity. The momentum, turbulent kinetic energy, specific dissipation rate and density were spatially discretized using the second order upwind scheme. The pressure was discretized using a PRESTO! scheme. A first-order implicit scheme was used to discretize the temporal term. The under-relaxation factors were 0.3, 0.7, 0.8 and 0.5 for pressure, momentum, energy, turbulence quantities, and density, respectively. Thirty iterations per time step were sufficient to attain the set convergence criteria of 10^-6^ for continuity and velocities. The turbulent quantities and vapour fraction were also converged to a minimum of 10^-4^ across all the cases. A customized field function (CFF) was written in Fluent to calculate the Euler Number with flow time.

(16) $$\mathrm{Eu}=\frac{\Delta P}{0.5\rho V_{T}^{2}}$$

The various geometries considered in this study were meshed using the Ansys Mosaic^TM^ meshing technology [27]. More than 95 per cent of the volume was filled with pure hex-core elements. A polyhedral boundary layer was used on the walls of the VD to capture the boundary layer to ensure that the value of y^+^ (y^+^ is the dimensionless distance from the surface of a solid object to a point within the adjacent fluid flow, calculated as $y^{+}=y\rho u_{*}/\mu$; where y is first cell centre height, ρ is density, $u_{*}$ is frictional velocity, where $u_{*}=\sqrt{\frac{\tau_{w}}{\rho }}$ and $\tau_{w}=C_{f}\frac{1}{2}\rho U_{f}^{2}$ $\tau_{w}$ is wall shear stress (Pa), $C_{f}$ is skin friction coefficient (-), $U_{f}$ is the free steam velocity (m/s), μ is dynamic viscosity [28] was maintained less than 5 for all the cases. The first height of the boundary layer was kept at 0.02 mm. The first height of 0.02 mm was calculated based on V_T_ = 2.3 m/s and for d_T_ = 12 mm to maintain the value of y+ ∼1. A total of 7 boundary layers were inserted with a growth rate of 1.4. The boundary layer was connected to the hex-core mesh with one layer of polyhedral elements. A body of influence (BOI) approach [29] was used to mesh the critical area of the VD geometry. By virtue of the geometry, the most critical area was the vortex chamber that hosts the main vortex that extends to the outlet duct of the VD’s geometry. Three BOIs, one covering the ∼ 75 % volume of the vortex chamber extending to 25 % of the outlet duct length and the other covering another 20 % of the outlet duct, were used. One more BOI was used to cover the outer half circular ring of the vortex chamber. The first BOI was meshed with a finer grid to capture the main vortex in the vortex chamber and its extension in the outlet duct. The size of BOI 1 to BOI 2 and 3, to the maximum size in the domain was increased by a factor of 2 with one peel layer in between. The second BOI was used as a buffer to ensure a smooth transition from the finer grid region of the BOI 1 to the BOI 2 after the course grid downstream of the outlet duct. Same sizing of BOI 2 was used for BOI 3 (Fig. S1 in the supplementary information shows the BOIs used in the fluid volume). All the critical attributes of the mesh, like orthogonal quality, aspect ratio and skewness, were kept within acceptable limits. The meshes at various critical locations for different VD configurations are shown in Fig. S2 of the supplementary information.

**Fig. 2:**
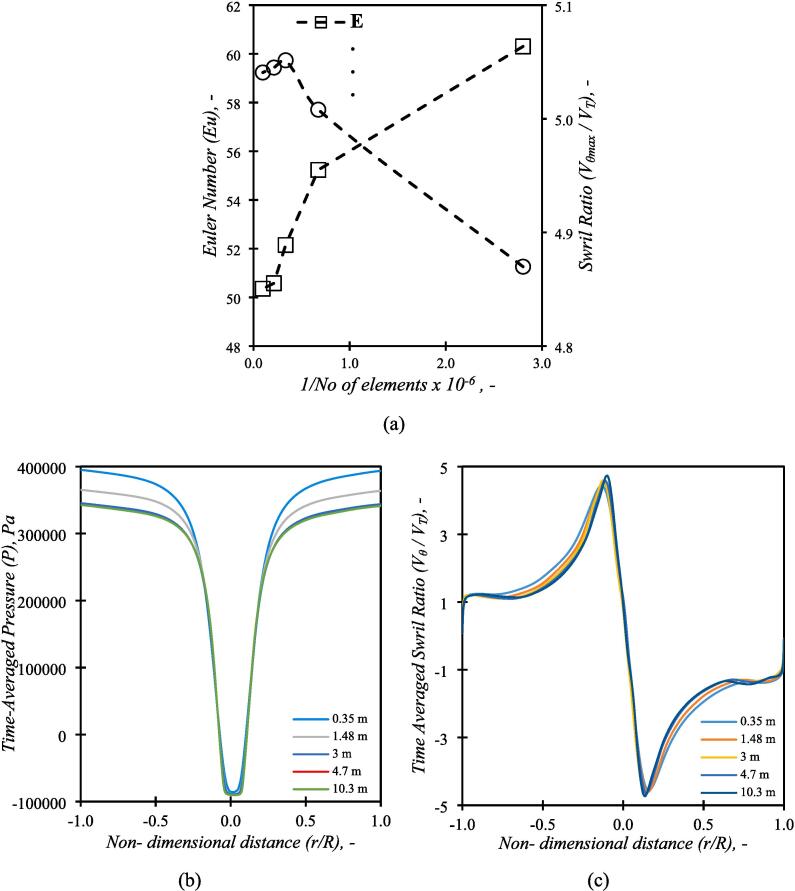
Grid Independence (a) Euler Number and Swirl ratio as a function of grid size, (b) Time-averaged pressure profile over mid line as a function of grid size, (c) Time-averaged swirl ratio profile over mid line as a function of grid size.

The grid independence was evaluated using five mesh sizes with 0.35, 1.48, 3, 4.7 and 10.3 million cells for a single inlet VD with$d_{T}$ = 12 mm and $D/d_{T}$ = 6 for the highest velocity, $V_{T}=3.9$ m/s. Influence of the number of computational cells on the simulated values of Euler number and the swirl ratio $\left(V_{\theta max}/V_{T}\right)$ are shown in Fig 2. The time-averaged pressure and the swirl ratio was also monitored over the midline in the vortex chamber. The time-averaging was initiated after 1τ of flow time. The time-averaged profiles of pressure and swirl ratio are shown Fig. 2b and 2c respectively. It can be seen that the difference between the predicted Euler number and the swirl ratio for 3 and 10.3 million cells was less than ∼ 3 %. In the interest of simulation time and demands on computational resources, subsequent simulations were carried out with 3 million computational cells. For other geometries with different D/d_T_ and scales, the same meshing strategy and parameter were used to mesh those geometries.

## 3 Experimental

**Fig. 3:**
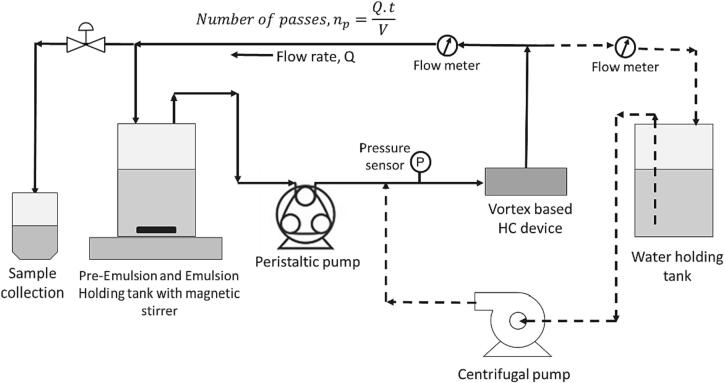
Schematic of experimental set-up for production of emulsions (solid line) and pressure drop versus flow rate (dashed line).

In this work, we experimentally investigated the VD's overall pressure drop and evaluated the performance based on the droplet size distribution of the emulsion. Fig. 3 shows the schematic of the experiment setup used for measuring overall pressure drop and production of emulsion. A peristaltic pump (Longer Model- BT600–2 J) and a centrifugal pump with a variable frequency drive (VFD) (pEDRELLO 3cr80) were used to pump the water through the VD. A ‘T’ junction was used near the inlet of the VD to facilitate the attachment of one end of a digital differential manometer (Digitron 2000P), the other end of the manometer was kept open to the atmosphere. An inline digital flowmeter (Krohne Magnetic Flowmeter – AF-E 400, https://krohne.com/en) was used to measure the flow rate in the fluid circuit. The RPM on the peristaltic pump was changed to change the inlet flow rate to the VD. Same set-up with water in the holding tank was used to measure pressure drop. A centrifugal pump was used to generate higher flows to cover the wider range of flow rates for pressure drop measurements and for large-scale of VD. Pressure readings were noted over a period of 5–7 min after the quasi-steady state was reached (after ∼ 5 min of turning ON the pump). The pressure readings were averaged to obtain the mean pressure drop across the VD. Three sets of readings were taken to ensure repeatability and to estimate the error bars. The overall pressure drop was determined for 12 mm single inlet, 3 mm single inlet, 3 mm two inlet and 3 mm simplified shape vortex based cavitation devices.

The tangential velocity profile over a midline in the vortex chamber of VD was measured with a laser Doppler anemometer (LDA, Dantec make https://www.dantecdynamics.com/). A 12 mm acrylic VD was used for this experiment. A centrifugal pump was used to pump water into the VD in a closed loop circuit. The flow rate into the VD was controlled by a variable frequency drive (VFD). An inline pressure sensor was also used to measure the pressure drop across the VD. A total of 36 points with an increment of 2 mm along the midline, were used to measure the tangential velocity in the vortex chamber. The measurement of the velocities was done at a depth of 6 mm inside the vortex chamber (mid-plane). Initially, the top and bottom walls of the VD were marked with the help of a traverse monitoring the anode current response in the BSA software (LDA data processing software by Dantec). Then the measurement volume/point of the LDA was moved inside the VD by 6 mm to reach the mid plane in the vortex chamber. The 6 mm depth for all the points were ensured by keeping the VD exactly perpendicular to the laser optics. Very small volume percent (≪1%) of hollow glass spheres of 10 µm were used as tracer particles (supplied by Dantec Dynamics). Settings were made in the BSA software to acquire data at each point for period of 20 s or for 10,000 data points, which ever was earlier. Three sets of readings at each point were taken over 3 sets of experiments to determine the error bars.

For the emulsions, a pre-emulsion was created using 5 % (by volume) rapeseed oil ($\rho_{o}=915$ kg/m^3^, $\mu_{o}=6.2\times 10^{-2}$ Pa.s) in demineralized water oil ($\rho_{o}=997$ kg/m^3^, $\mu_{o}=6.2\times 10^{-2}$ Pa.s) and 2 % wt TWEEN 20 was used as surfactant. This was same composition used by Thaker and Ranade [7]. A total volume of 500 mL pre emulsion was used. The oil-in-water pre-emulsion was generated by adding 5 % (by volume) of oil volume in water and using a magnetic stirrer operated at 350 rpm for 10 min following Thaker and Ranade [7]. The pre-emulsion was then pumped through the VD via a peristaltic pump (Longer BT600–2 J). The schematic of the experimental for emulsion production is given in Fig. 3. A 3 mm single inlet standard and a 3 mm simplified vortex based devices were used for the emulsion productions. The emulsion samples obtained at passes 1, 5, 10, 30, 50, 100 and 200 were analysed using a Malvern MasterSizer 3000. The measurements were performed by using water as a dispersant medium and setting the refractive index for rapeseed oil as 1.466 [7].

## 4 Results and discussion

For applications involving the use of VD harnessing its ability to cavitate, the performance parameters needs to be defined appropriately. The designs may be compared based on the strength of the vortex generated and subsequent extent of cavitation as well as overall pressure loss (in other words, energy consumption). The strength of vortex generated in the device impacts the minimum pressure value in the device which defines the inception of cavitation and influences the extent of cavitation. The maximum tangential velocity, $V_{\theta max}$ or the swirl ratio ($V_{\theta max}/V_{T}$*)* is used to characterise the strength of the vortex in the device. The extent of cavitation may be related to the amount of vapor generated in the device. In order to define a cavitation performance parameter across different scales, a ratio of vapour volume to device volume was used. The cavitation performance parameter, $C_{E}$, is define as:

(17) $$C_{E}=\left(\frac{V_{\mathrm{vap}}}{V_{D}}\right)\left(\frac{1}{\Delta PQ}\right)$$

Here, $V_{\mathrm{vap}}$ is the volume of vapor generated in the device and $V_{D}$ is the volume of the vortex based device. ΔP is pressure drop across the device and Q is flow rate through the device. Pressure drop versus flow through the device relationship is characterised by the Euler number (Equation (16)). The performance parameter $C_{E}$ could be used to gauge the extent of cavitation achievable in a particular device for the given set of operating conditions. The cavitating performance of the device is the ratio of (number of cavities/s)/ (flow rate, m^3^/s) which is essentially number of cavities per m^3^ of processed fluids per unit power consumption.

As discussed in previous studies, a strongly swirling flow sets up in the vortex based devices considered in this work. A classical model for such confined vortex flows is based on a combination of forced vortex in the vortex core surrounded by a free vortex. The transition from free to forced vortex is a region of maximum tangential velocity. The Rankine vortex model is applicable for a purely inviscid swirl flow made up of a core of radius, $r_{c}$, rotating as solid body:

(18) $$V_{\theta }=\;\left\{\;\begin{array}{c} \frac{\Gamma }{2\pi r_{c}^{2}}\;\;\;\;\;\;\;\;\;\;\;\;\;\;r0\leq r\leq r_{c}\\ \frac{\Gamma }{2\pi r}\;\;\;\;\;\;\;\;\;\;\;\;\;\;r_{c}\leq r\leq \infty \end{array}\right)$$

Here, Γ is the circulation of the vortex. Based on this Rankine vortex model, the maximum tangential velocity may be written as:

(19) $$V_{\theta max}=\frac{\Gamma }{2\pi r_{\max}}$$

In the devices considered in this work, the location of maximum tangential velocity is close to $0.5d_{T}$. The circulation can therefore be written in terms of maximum tangential velocity and inlet velocity at the periphery of the device (at r=0.5D). The inlet diameter is same as the throat diameter and therefore inlet velocity is same as the throat velocity ($V_{T}$).

(20) $${\Gamma =\pi V}_{\theta max}d_{T}={\pi V}_{T}D$$

Thus, for a Rankine vortex model the relation for swirl ratio as a function of aspect ratio ($D/d_{T}$) is obtained as: $\frac{V_{\theta max}}{V_{T}}=\frac{D}{d_{T}}$. This indicates that as aspect ratio increases, swirl ratio increases. It should however be noted that this is based on the assumption that the transition of free vortex to forced vortex occurs at throat radius. This assumption is reasonable for inviscid or ideal flows. In practical confined vortex flows, the location of the transition point (or maximum tangential velocity) is influenced by viscous effects as well as the wall effects confining the flow. Secondly the Rankine model neglects the effects of friction which may become important in confined vortex flows. Therefore, in this work, we used computational flow model to understand influence of aspect ratio.

Initially, the base case was chosen with scale$d_{T}$ = 12 mm and$D/d_{T}$ = 6. The key results of the computational model (pressure loss and tangential velocity profiles) are compared with the experimental data (see Section 4.1). The validated model was then used to investigate influence of various design parameters. These results are discussed in Sections 4.2 onwards.

### 4.1 Validation of the computational model

The simulated results for the base case using the presented computational model are compared with the experimental data in Fig. 4. The comparison of the experimental and the simulated pressure drop for a range of throat velocities is shown in Fig. 4a. It can be seen that the CFD model was able to simulate experimentally observed pressure drop across the considered cavitation device over the range of flow rates. It is important to verify whether the CFD model is able to capture establishment of a strongly swirling flow in such devices when operated in a cavitating regime. The tangential velocity profiles were measured for three values of the throat velocity viz 1.95, 2.32 and 2.93 m/s corresponding to 100 kPa, 150 kPa and 250 kPa pressure drop across the device respectively. Fig. 4b shows the comparison of simulated and experimental radial profiles of dimensionless tangential velocity (swirl ratio) over the midline in the vortex chamber. The simulated swirl ratio profiles are in good agreement with the experimental data indicating that the simulation methodology is sufficient to capture the key features of flow in vortex based cavitation device. As stated earlier, rigorous validation was not performed, as the major focus of this work was to use the computational model for comparing different designs of the cavitation device. More rigorous validation of cavitation and turbulence models is in progress and will be published separately.

**Fig. 4a:**
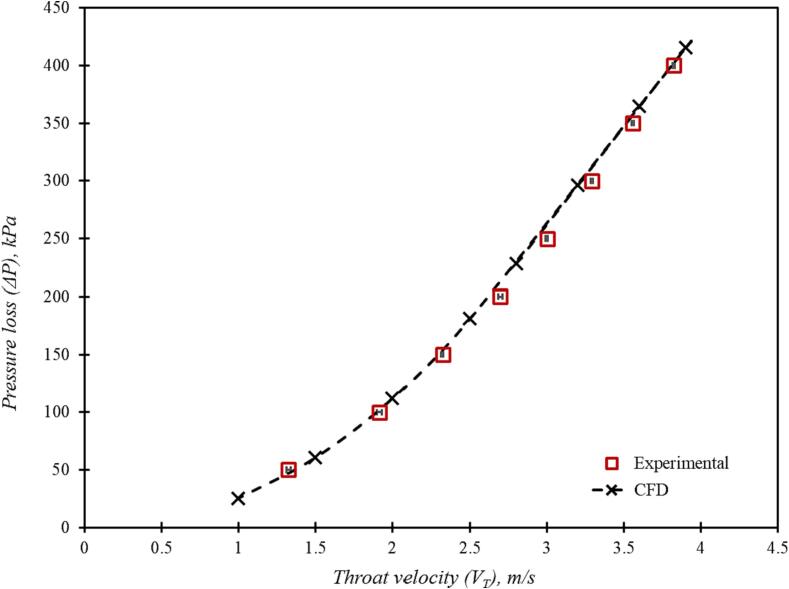
Comparison of experimental and simulation pressure loss (ΔP) versus throat velocity ($V_{T}$) data.

**Fig. 4b:**
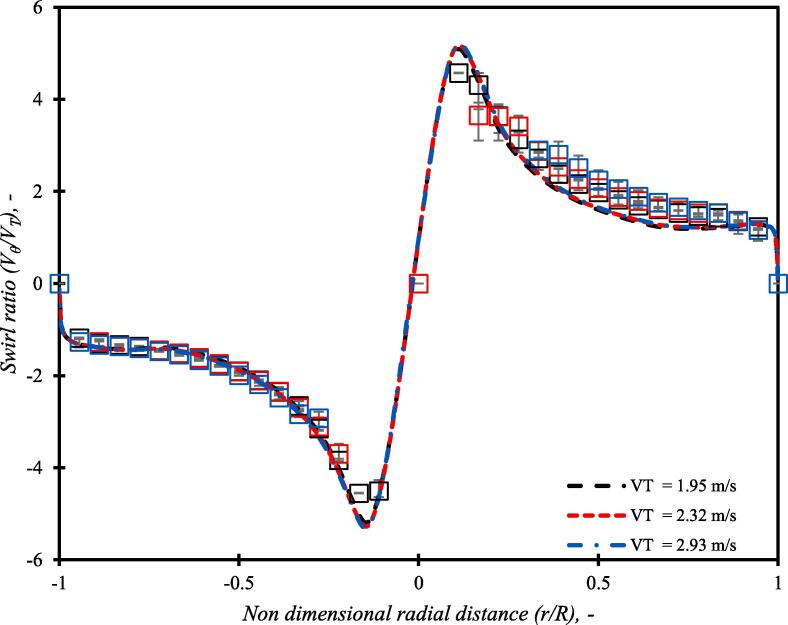
Comparison of experimental and simulated swirl ratio over the midline in vortex chamber (the dotted line represents CFD results while symbols represent LDA results).

**Fig. 5:**
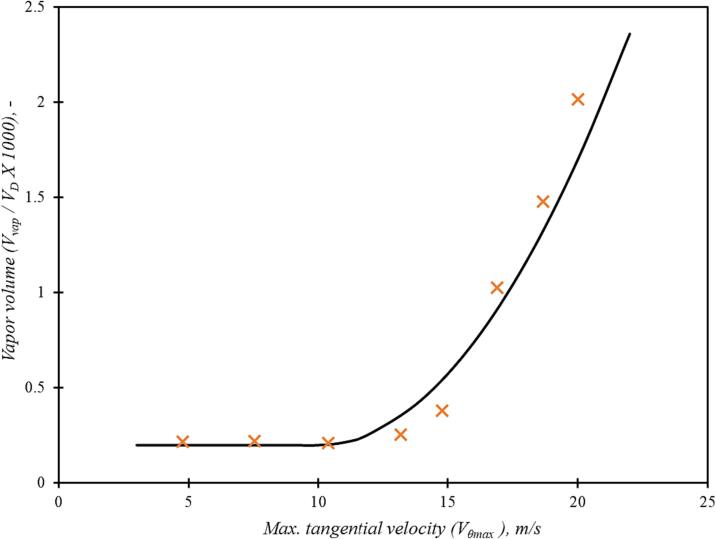
Variation of non-dimensional vapor volume with maximum tangential velocity for scale, $d_{T}$ = 12 mm; aspect ratio, $D/d_{T}$ = 6.

For examining the extent of cavitation, the simulated variation of non-dimensional vapor volume ($V_{\mathrm{vap}}/V_{D}$) for the base case is shown in Fig. 5. Since cavitation occurs in the core region, the amount of vapour generated in the cavitation device ($V_{\mathrm{vap}}$), is calculated by integrating the vapour volume in the core region (defined as cylinder of diameter $d_{T}$ with a length of three times $d_{T}$ stretching from the bottom surface of the vortex chamber towards axial outlet). It can be seen that as the maximum tangential velocity increases beyond 10 m/s (corresponding to pressure drop across the device about 100 kPa), the vapour generation increases rapidly with further increase in velocity (flow rate). There is a finite vapour volume below this inception limit which is because of the non-zero value of non-condensable gases specified in the cavitation model used here (the cavitation model of Singhal et al., [26]). This indicates that cavitation inception occurs when maximum tangential velocity approaches ∼ 10 m/s (or pressure drop crosses ∼ 100 kPa). This is in agreement with the previously published studies on inception of cavitation in such vortex based devices [1]. The validated computational model was used to examine influence of key design parameters.

### 4.2 Influence of aspect ratio ($D/d_{T}$) and scale ($d_{T}$)

**Fig. 6:**
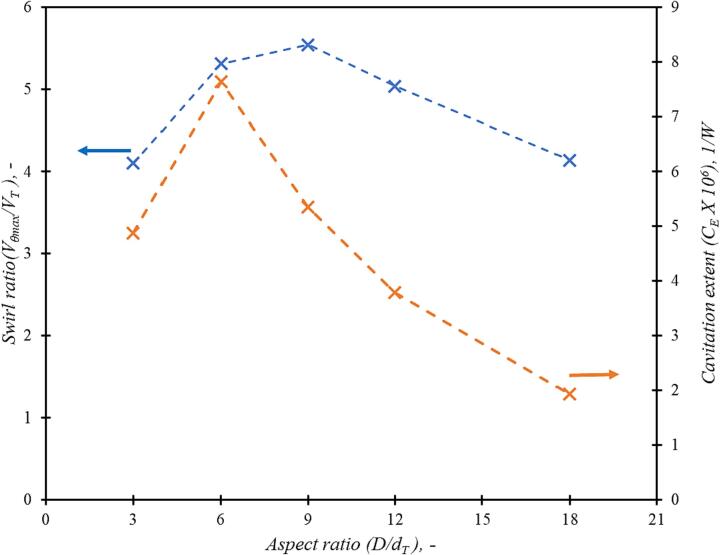
Influence of aspect ratio (*D/d_T_*) on swirl ratio and cavitation extent (C_E_) for throat velocity, *V_T_* = 2.93 m/s and *d_T_* = 12 mm.

The influence of aspect ratio on the performance of the vortex based device is discussed for base case of scale *d_T_* = 12 mm. The base case simulations were performed for throat velocity of 2.93 m/s for aspect ratio varying as 3, 6, 9 and 12. The throat velocity value 2.93 m/s corresponds to 250 kPa of pressure drop for base case scale *d_T_* = 12 mm and aspect ratio *D/d_T_* = 6. Previous study by Thaker and Ranade [9] was performed with a similar base case; hence, same case was chosen as the base case. The analysis based on the Rankine vortex indicates that maximum tangential velocity or maximum swirl ratio will monotonously increase with the chamber aspect ratio (*D/d_T_*). However, CFD results indicate that the swirl ratio increases with increase in aspect ratio, reaches a maximum value around aspect ratio value of 6 and then decreases again with further increase in aspect ratio (see Fig. 6).

It can be seen from Fig. 6 that the swirl ratio at aspect ratio ($D/d_{T}$) = 6 and 9 are very close (within 5 %). The maximum swirl ratio decreases when ($D/d_{T}$) < 6 or ($D/d_{T}$) > 9. The cavitation performance parameter, C_E_, is highest at aspect ratio of 6. It can therefore be concluded that aspect ratio of 6 ($D/d_{T}$ = 6) is an optimum aspect ratio. This is in agreement with earlier studies on non-cavitating flows by Priestman [10] and Kulkarni et al., [5].

**Fig. 7:**
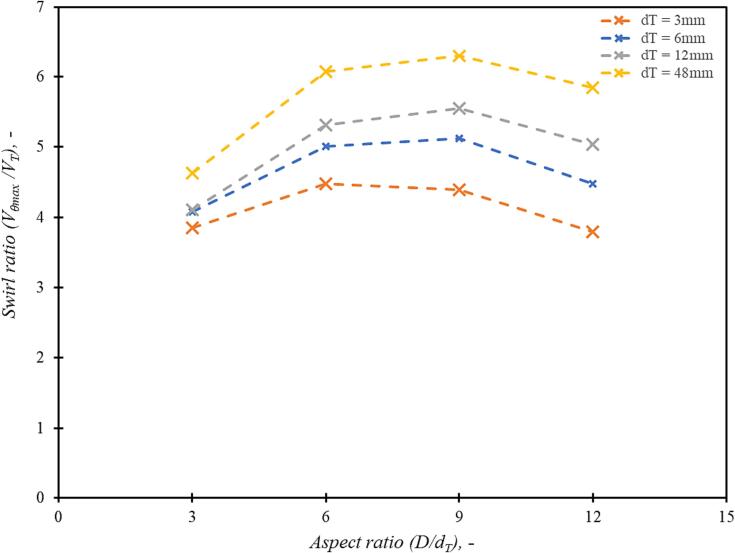
Comparison of maximum tangential velocity on centreline for different values of aspect ratio ($D/d_{T}$) at constant throat velocity, $V_{T}$ = 2.93 m/s and at scale $d_{T}$ = 3, 6, 12 and 48 mm.

In order to examine potential influence of device scale on the optimum value of aspect ratio, further simulations were carried out for devices of different scales. CFD simulations were therefore carried out for devices with throat diameters ranging from 3 mm to 48 mm for aspect ratio values ranging from 3 to 12. The simulated values of maximum swirl ratio are shown in Fig. 7. It can be seen that the behaviour is almost the same for all the device scales considered here (with more than 250 times scale-up in nominal capacity). These results indicate that the optimum value of aspect ratio may be considered as 6 irrespective of device scale.

**Fig. 8:**
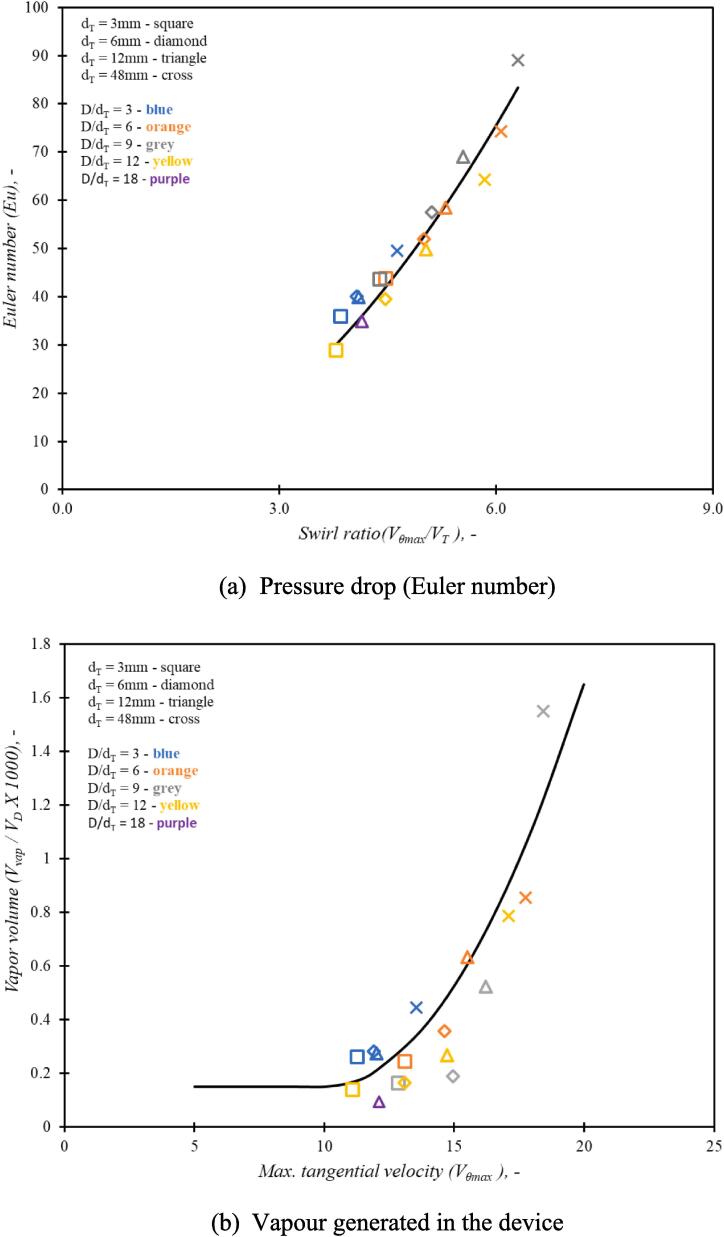
Simulated pressure drop and vapour generated at$V_{T}$ = 2.93 m/s for devices with aspect ratio ($D/d_{T}$) = 3,6, 9 and 12 and scale ($d_{T}$) = 3, 6, 12 and 48 mm. Line is Equation (22).

Influence of scale on simulated values of pressure drop (Euler number) and extent of cavitation is shown in Fig. 8a and 8b respectively. It can be seen from Fig. 8a that the Euler number, Eu when plotted as a function of swirl ratio, $V_{\theta max}/V_{T}$, the simulated results across all the aspect ratios and all the scales considered here follow almost parabolic relationship as:

(21) $$\mathrm{Eu}\propto {\left(V_{\theta max}/V_{T}\right)}^{2}$$

The proportionality constant is about 2. The variation of non-dimensional vapor volume ($V_{\mathrm{vap}}/V_{D}$) with maximum tangential velocity ($V_{\theta max}$) for different values of aspect ratio and scale are shown in Fig. 8b. It can be seen that all the cases considered in this work show similar behaviour exhibiting significant increase in vapour generation when maximum tangential velocity increases beyond ∼ 10 m/s. The dimensionless vapour volume also exhibits a parabolic relationship with maximum tangential velocity (beyond a critical value required for inception of cavitation) as:

(22) $$\frac{V_{\mathrm{vap}}}{V_{D}}1000=a{\left(V_{\theta max}-V_{\theta c}\right)}^{2}+c$$

The critical tangential velocity $\left(V_{\theta c}\right)$ or maximum tangential velocity for cavitation inception is seen to be around 10 m/s, and the values of constants a and c are 0.015 and 0.15 respectively.

**Fig. 9:**
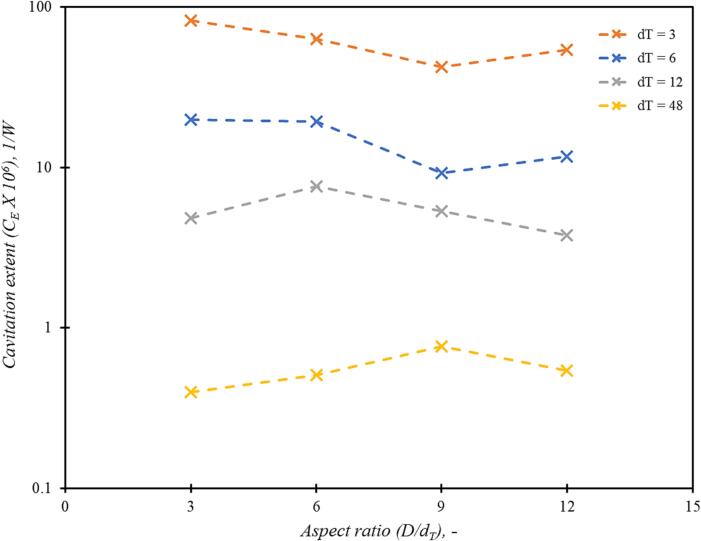
Influence of aspect ratio ($D/d_{T}$) on the extent of cavitation for constant throat velocity $V_{T}$ = 2.93 m/s and at $d_{T}$ = 3, 6, 12 and 48 mm.

The effective cavitation performance of the device may be characterised by the previously defined cavitation performance parameter ($C_{E}$). Simulated values of cavitation performance parameters are shown in Fig. 9. It can be seen from Fig. 9 that increase in device scale reduces the cavitation performance parameter. This observation is in agreement with the previously published results of influence of scale on drop breakage (Thaker and Ranade [7]) and on degradation of organic pollutants in water (Ranade et al., [17]).

Based on the results presented in this section, it may be concluded that the chamber aspect ratio of six ($D/d_{T}$ = 6) and the device with throat diameter $d_{T}$ of 3 mm give the best performance among the considered devices. This configuration was therefore selected for further investigations.

### 4.3 Influence of the number of inlets ($n_{I}$)

**Fig. 10:**
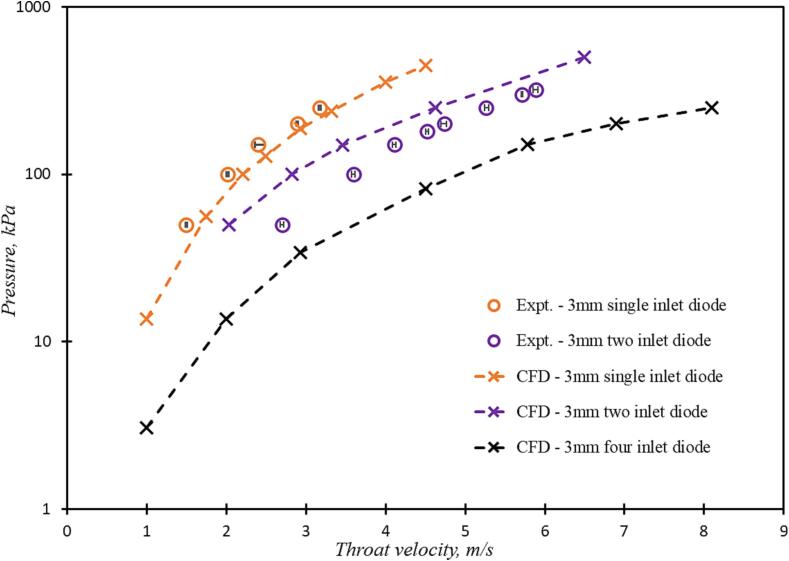
Comparison of experimental and simulation pressure drop for one, two and four inlet devices ($d_{T}=3mm$); Experimental data is shown as circles and CFD results are shown as dashed lines with cross symbols.

Influence of tangential inlet on the flow characteristics of the vortex based cavitation devices was examined by simulating flow with one, two and four tangential inlets (see Fig. 1b). The simulated values of pressure drop as a function of throat (at the outlet of the device) velocity is shown in Fig. 10. The experimental data obtained for the one and two inlet devices is also included in Fig. 10. The discrepancies in the simulated and experimental results may be attributed to small variation caused due to manufacturing tolerances and gasket thickness. Considering the smaller size of the device ($d_{T}$ of 3 mm), a small difference in the chamber depth caused by the gasket (∼10^2^ µm) may cause such a difference. Despite the discrepancies, it may be said that the simulated results capture the trends in the experimental data reasonably well. It can be seen from Fig. 10 that increase in number of inlets decreases pressure drop across the device for the same flow rate (for the same velocity at the outlet throat of the device).

**Fig. 11:**
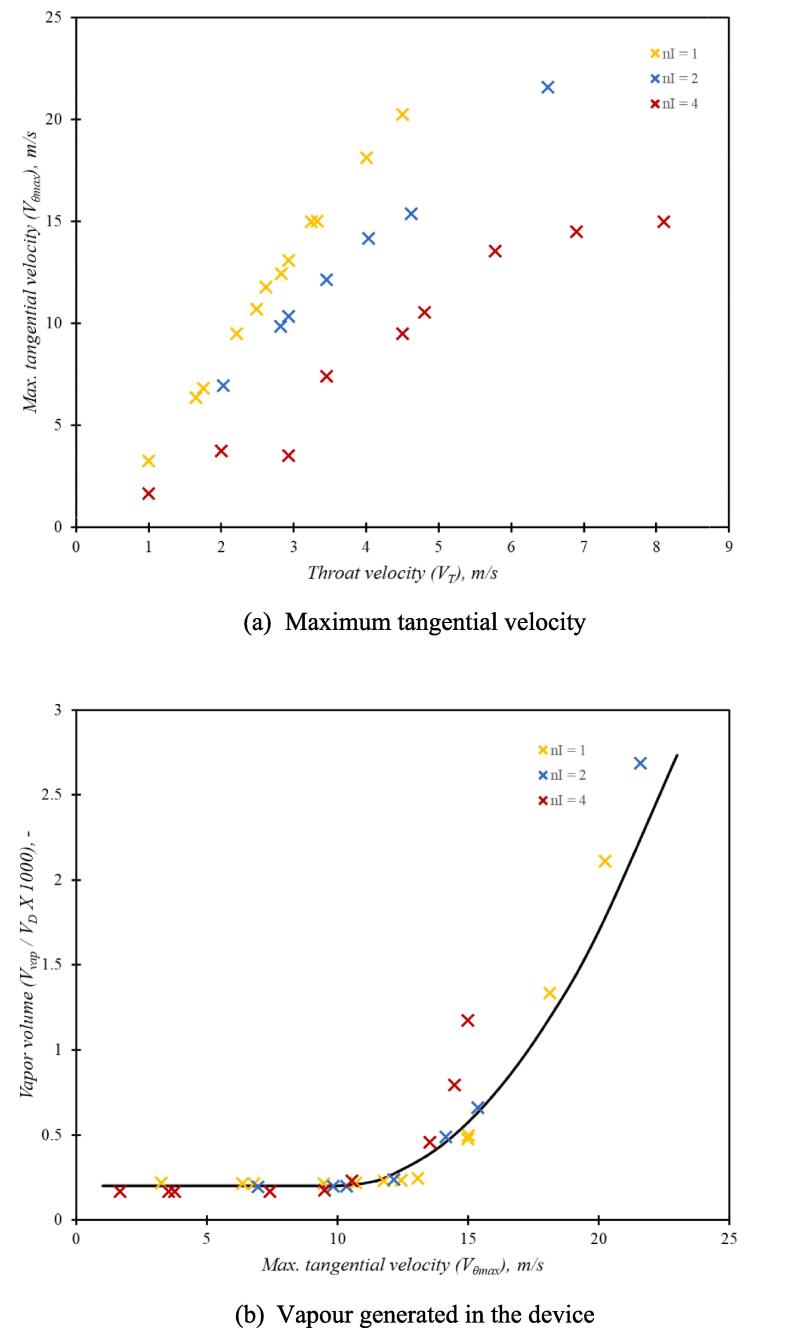
Influence of number of inlets maximum tangential velocity and vapour generation.

The Euler number for the one, two and four inlet devices was seen to follow relationship given by Equation (21). The effect of increasing the number of inlets on maximum tangential velocity and vapour generated is shown in Fig. 11a and 11b respectively. It can be seen that as number of inlets increase, maximum tangential velocity decreases for the same flow rate through the device (same value of outlet throat velocity). This is expected since with increase in number of inlets, the velocity at the inlet throat is reduced. This reduction in maximum tangential velocity causes reduction in observed pressure drop across the device as number of inlets increase (as seen in Fig. 10). Since the multiple inlet devices were found to follow Equation (21), it can be further inferred that for devices operating at same pressure drop values, the maximum tangential velocity obtained is similar and independent of the number of inlets. The dimensionless vapour generated in multiple inlet devices exhibited behaviour similar to those of a single inlet device (see Fig. 11b). The results indicate that the Equation (22) is valid even for multiple inlet devices with critical maximum tangential velocity as ∼ 10 m/s. From Fig. 11b, it is observed that the maximum tangential velocity required for cavitation inception decreases to some extent with an increase in the number of inlets. For a particular value of maximum tangential velocity ($V_{\theta max}$), the four inlet device is seen to perform somewhat better than a one or two inlet device. This increase in cavitation extent is a result of increased flow rates for the same pressure loss in the four inlet device as compared to a one inlet or two inlet device. Considering the similar performance and the ease of fabrication, single inlet device is selected for further investigation towards facilitating numbering up/ scale-out approach.

### 4.4 Simplified configuration of vortex based cavitation device

Considering the adverse impact of scale-up on device performance, an option of scale-out or number-up looks promising. With the scale-out approach, higher throughput may be obtained by simply using multiple devices. An effective way of numbering-up is to suitably integrate multiple units to facilitate fabrication of a set of desired number of devices in one go. The geometry of the vortex based devices considered so far is rather complex involving difficult to fabricate connections of vortex chamber with inlet and outlet. Numbering up of such a device is not straightforward. Therefore, in this work, we evaluated potential simplifications of the device configuration without jeopardising the performance. Based on the results obtained so far, we used the device with $d_{T}$ = 3 mm, $D/d_{T}$= 6 and $n_{I}$ = 1 for potential simplifications. We primarily focussed on simplification of following features:

• Curvature at the periphery of the chamber
• Profile of the outlet port
• Profile of the inlet port

Based on preliminary trials, we eventually developed a simplified configuration of device by simplifying the vortex chamber as cylindrical chamber with depth of $d_{T}$. The complex profile of the outlet port was simplified to a cylindrical one with diameter of $d_{T}$. The taper of the inlet port was also eliminated. The cross-section of the inlet port was modified from circular to square which facilitates fabrication. With these simplifications, it was verified that integrated assembly of multiple devices can be manufactured. Before manufacturing integrated multiple devices, it was essential to evaluate the impact of simplified configuration on performance of the device, which is discussed here.

**Fig. 12:**
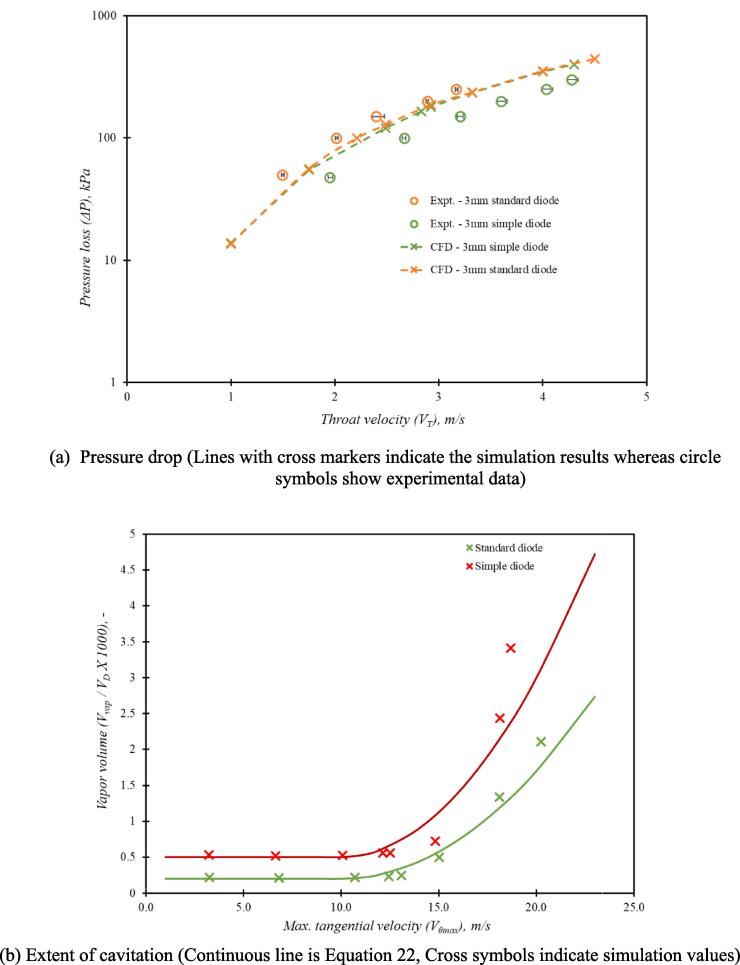
Comparison of simplified and standard configurations of vortex based cavitation devices with *d_T_* = 3 mm and *D/d_T_* = 6.

A vortex based cavitation device based on the simplified geometry (see Fig. 1c) was fabricated and tested. Comparison of simulated and experimental pressure drop obtained with the standard and simplified configuration of vortex based devices is shown in Fig. 12a. It can be seen that simplifying the device design does not impact the pressure drop versus flow rate behaviour significantly. This is expected as the major pressure loss occurs near the vortex core region which is retained while simplifying the design. The comparison of simulated tangential velocity profiles of standard and simplified device also shows similar behaviour. The extent of vapour generated as a function of maximum tangential velocity for the simplified and standard device is compared in Fig. 12b. It can be seen that both the devices follow a similar trend which is consistent with the Equation (22). In case of a simplified configuration, the critical value of maximum tangential velocity for inception remains around 10 m/s, the values of parameters a and c of Equation (22) are seen to increase to 0.025 and 0.5 respectively. This implies that simplifying the device configuration does not impact the performance significantly and hence could be used as an option for scale-out.

**Fig. 13:**
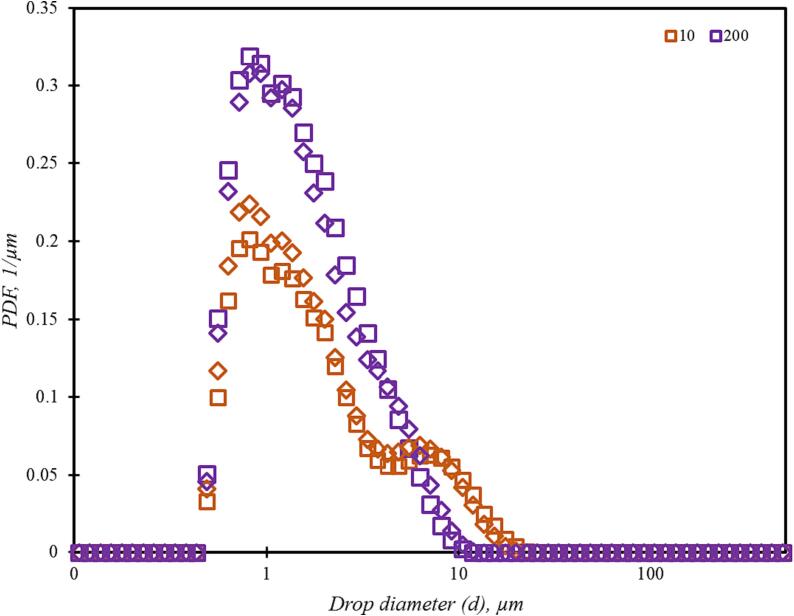
Comparison of DSD for standard and simple vortex based device. Square symbols are for simplified configuration and diamond symbols represent data for standard configuration. Colour indicate number of passes.

In order to verify this conclusion of nearly same performance of the standard and simplified device, a case of liquid–liquid emulsion was considered. The use of vortex based hydrodynamic cavitation devices for producing emulsions has been undertaken by many researchers and offers an effective alternative to conventional techniques [1], [30]. The performance of the simplified and standard devices is compared in terms of obtained droplet size distribution (DSD), Sauter mean diameter ($d_{32}$) and breakage efficiency (η). The results of the standard device design are taken from a previous publication by Thaker and Ranade [7]. The experiments were performed as discussed in Section 2.2. The comparison of DSD obtained with simplified device is compared with that obtained with the standard device in Fig. 13. It can be seen that the difference in the DSDs obtained with the two devices is small and decreases as the number of passes through the device increase.

The Sauter mean diameter and droplet breakage efficiency were calculated using the standard procedure [7] as:

(23) $$d_{32}=\frac{\sum_{i=1}^{N_{B}}d_{i}^{3}{f\left(d_{i}\right)dd}_{i}}{\sum_{i=1}^{N_{B}}d_{i}^{2}{f\left(d_{i}\right)dd}_{i}}$$

(24) $$\eta =\frac{E_{m}}{\Delta PVn_{p}}=\frac{6\alpha_{0}\sigma }{\Delta Pn_{p}}\left[{\left(\frac{1}{d_{32}}\right)}_{n_{p}}-\frac{1}{d_{320}}\right]$$

**Fig. 14:**
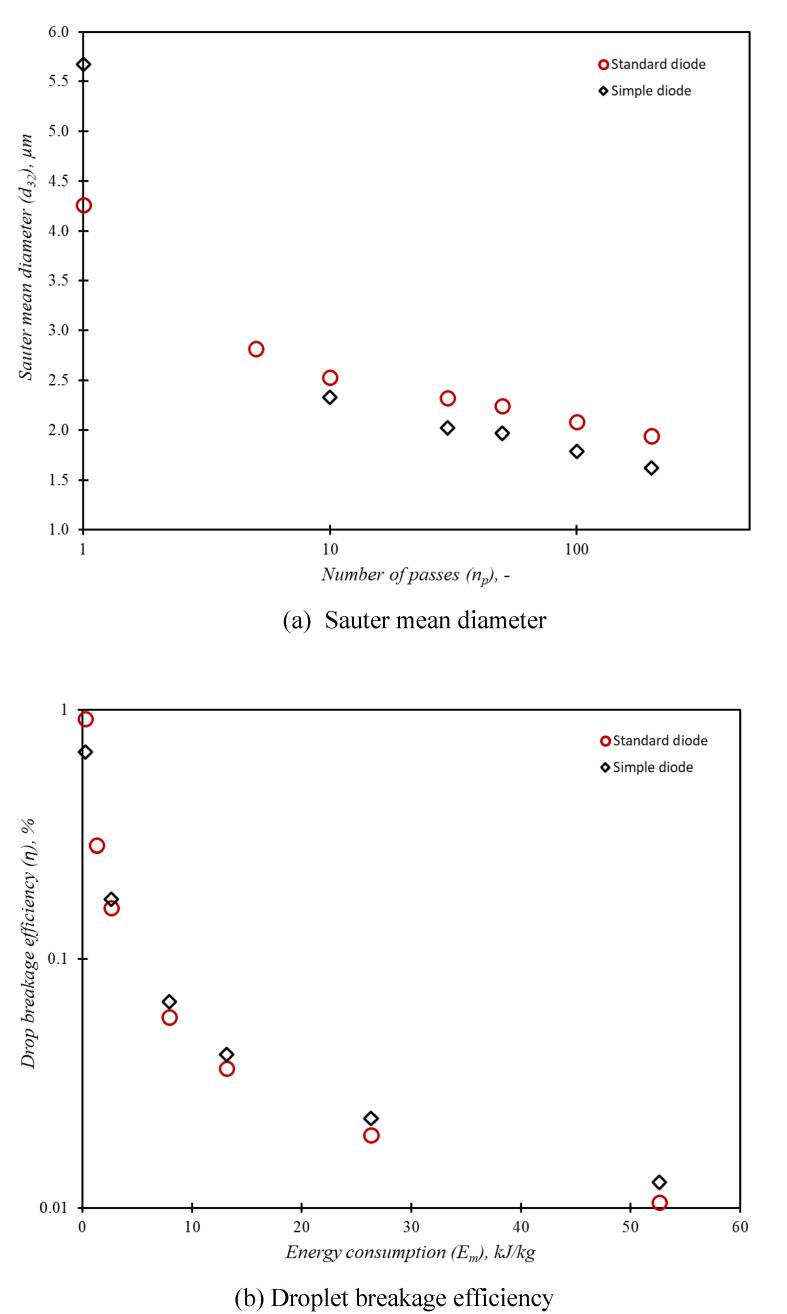
Comparison of emulsions generated by simplified and standard vortex based devices (*d_T_* = 3 mm). Red circles: simplified device; Black diamonds: standard device.

Individual symbols are explained in the nomenclature section. The comparison of Sauter mean diameter and droplet breakage efficiency as a function of number of passes for the simplified and standard device is shown in Fig. 14a and 14b respectively.

It can be seen that for number of passes more than ten, the performance of simplified and standard devices is almost the same. The results presented in this work will be useful to select optimum configuration of vortex based cavitation devices/ reactors for a variety of applications beyond liquid–liquid emulsions.

## 5 Conclusions

The design of vortex based cavitation devices is investigated using multiphase CFD simulations. The simulation methodology is validated by comparing simulated results of pressure drop and tangential velocity profile for a base case of cavitation device. Validated computational model was then used to evaluate influence of aspect ratio of vortex chamber, number of tangential inlets and shape of the device on flow and performance parameters. The performance of the recommended design was evaluated by considering a case of production of liquid–liquid emulsion. The key findings of this work are summarised in the following.

• For all the configurations and operating conditions of vortex based cavitation devices considered in this study, the dimensionless pressure drop (Euler number, Eu) was found to be directly proportional to the square of swirl ratio ${\left(V_{\theta max}/V_{T}\right)}^{2}$ with the constant of proportionality as ∼ 2.
• For all the cases considered here, the dimensionless vapour volume ($V_{\mathrm{vap}}/V_{D}$) was found to rise sharply beyond a critical value of maximum tangential velocity ($V_{\theta c}$). The value of $V_{\theta c}$ was found to be ∼ 10 m/s. Beyond this critical value, the dimensionless vapour volume ($V_{\mathrm{vap}}/V_{D}$) was found to be proportional to the square of difference between maximum tangential velocity and $V_{\theta c}$.
• The cavitation performance parameter, $C_{E}$ (defined by Equation (17), was found to be useful for comparing performance of different device designs and operating conditions.
• The results of influence of aspect ratio and scale on the performance of single inlet device indicate that out of the considered cases, the smallest device with $d_{T}=3mm$ with aspect ratio of six ($D/d_{T}=6$) was found to be the best.
• Increase in number of inlets was found to reduce pressure drop across the device for the same flow rate with corresponding decrease in the maximum tangential velocity. For the same pressure drop across the device, the maximum tangential velocity generated in the device is independent of the number of inlets. Correspondingly, the performance of the devices for the same pressure drop is also similar. These results indicate that performance of multiple units of single inlet device would perform better than single device with multiple inlets.
• For facilitating integrated manufacturing of multiple units of single inlet device, appropriate simplifications in the device configuration are proposed. The simulated results of the proposed simplifications indicated that the simplified device exhibited similar performance in terms of maximum tangential velocity as well as volume of vapor generated in the device to the standard configuration of the device. This was verified by comparing DSD, Sauter mean diameter and breakage efficiency for oil in water emulsions. Beyond ten passes through the devices, the performance of the standard and simplified devices was found to be comparable.

The presented approach and results will be useful for selecting and optimising design of vortex-based cavitation devices/ reactors for variety of applications.

## CRediT authorship contribution statement

**Amol Gode:** Investigation, Data curation, Validation. **Ketan Madane:** Investigation, Data curation, Validation. **Vivek V. Ranade:** Conceptualization, Funding acquisition, Supervision.

## Declaration of Competing Interest

The authors declare that they have no known competing financial interests or personal relationships that could have appeared to influence the work reported in this paper.

## Data Availability

Data will be made available on request.
