# Supplementary material for: Design of vortex-based cavitation devices/reactors: Influence of aspect ratio, number of inlets and shape
Source: Ultrason Sonochem. 2023 Nov 22;101:106695. doi: 10.1016/j.ultsonch.2023.106695 (PMC10767635; doi:10.1016/j.ultsonch.2023.106695)
Supplement: Supplementary data 1 [file mmc1.pdf]

Supplementary Information

Design of Vortex-based Cavitation Devices/ Reactors:
Influence of aspect ratio, number of inlets and shape

Amol Gode, Ketan Madane and Vivek V. Ranade
Multiphase Reactors and Intensification Group (mRING)
Bernal Institute, University of Limerick, Ireland
*Corresponding Author: Vivek.Ranade@ul.ie

S1. **Use of URANS SST k-ω turbulence model**

Simpson and Ranade [Simpson, A. and V.V. Ranade, *Flow characteristics of vortex based cavitation devices. AIChE Journal*, 2019. **65**(9)] have discussed the selection of the turbulence model for simulating cavitating flow in a vortex based hydrodynamic cavitation device. It is emphasized that the selection of appropriate turbulence closure model is complicated due to the presence of interaction between turbulence and secondary phase generated due to cavitation. The selection of appropriate turbulence model will lead to accurate prediction of tangential velocity profile which further translates to accurate prediction of pressure profiles realising cavitating conditions. The turbulence models evaluated were, Reynolds stress model (RSM), RNG k-ε, SST k-ω and SST k-ω with Menter, and Kato and Launder turbulence production limiting functions. The standard k-ε turbulence model over-predicted the turbulent kinetic energy leading to the lower tangential velocities. The results from using SST k-ω with production limiters led to a closer agreement with RSM. The use of RSM requires significantly more computational resources (due to increase in number of equations being solved). Based on these conclusions from the study of Simpson and Ranade, it was decided to use the URANS SST k- ω turbulence model with turbulence production limiter functions which do not require undue computational resources without jeopardising the accuracy.

S2. **Key aspects of the computational model**

The table below summarizes the key aspects of the computational model and rationale behind these:

**Aspect**	**Selected option**	**Rationale**
**Approach of modelling Turbulence**	Selected unsteady Reynolds Averaged Navier Stokes (URANS) approach for turbulent cavitating flows.	Large eddy simulations of full three-dimensional device with phase change were computationally too demanding. URANS approach was shown to capture key details of the cavitating flows and therefore adopted in this work.
**Turbulence model**	SST k-ω model	See Section S1.
**Cavitation model**	Singhal model [Singhal Ashok K., A.M.M., Li Huiying, Jiang Yu, Mathematical basis and validation of the full cavitation model. *Journal of Fluids Engineering*, 2002. **124**: p. 617-624]	The cavitation model is based on the Rayleigh-Plesset equation for vapor bubble growth and collapse in a liquid. The effect of presence of non-condensable gases is considered and assumed to be known constant. The model has shown to be effective for different cavitating flows. It was also used successfully by Simpson & Ranade for vortex based cavitation devices.
**Boundary conditions**	Inlet: Velocity inlet<br>Outlet: Pressure outlet<br>Walls: no-slip	The combination of velocity inlet and pressure outlet boundary conditions provides a stable solution.
**Solution domain**	Extended axial port length to 50 times the throat diameter	To avoid backflow at the outlet.

S3. **Mesh details**

The Figure S1 gives the BOI location with respect to the domain. The images of the generated mesh are shown in Figure S2.

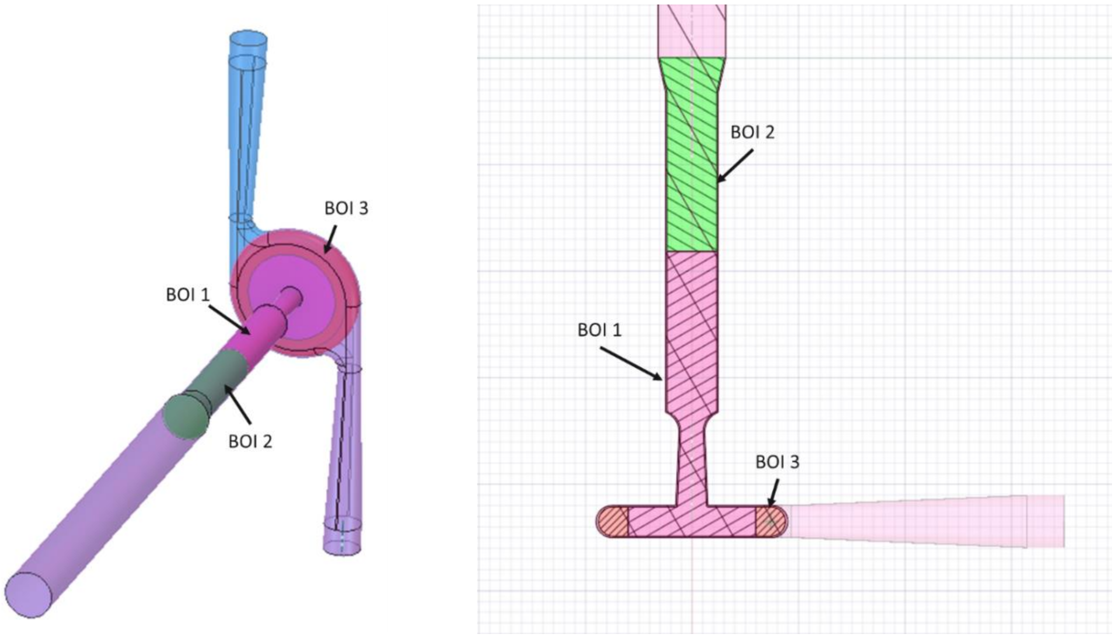

(a) (b)

Figure S1. Location of BOI 1, BOI 2 and BOI 3 in the computational domain.

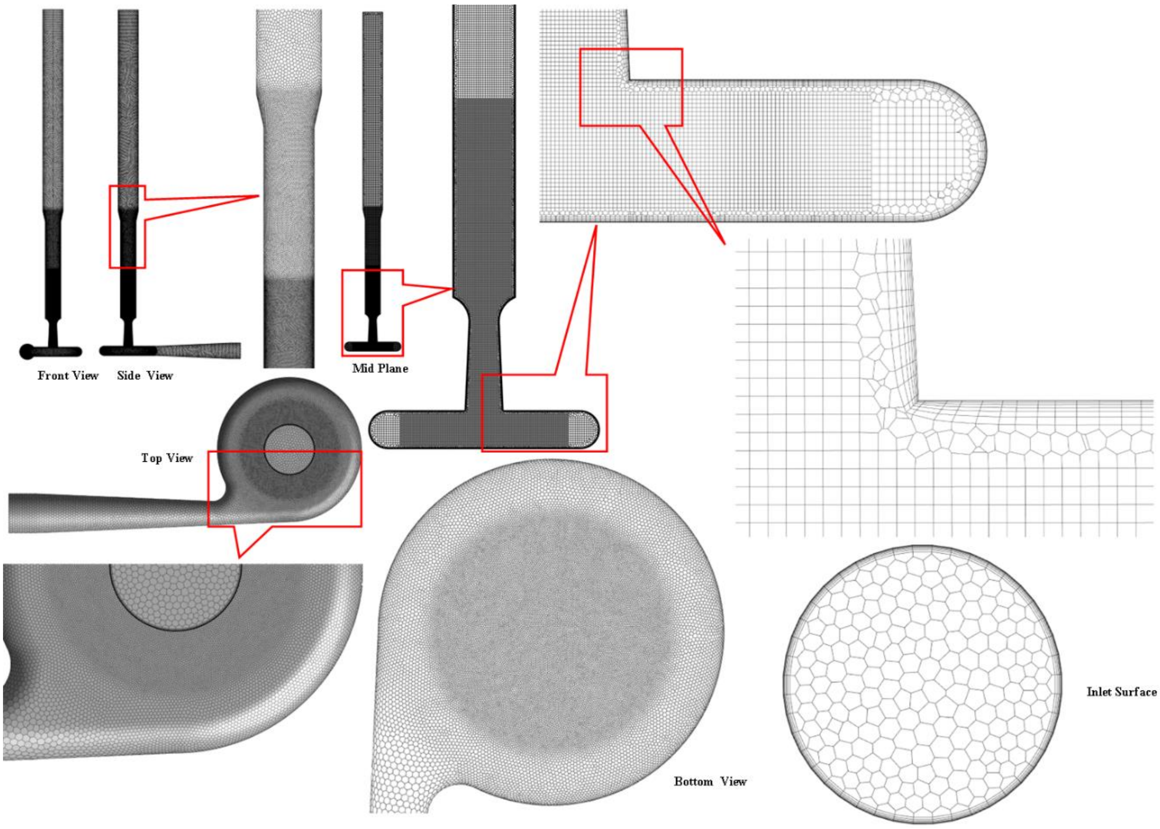

Figure S2(a). Mesh at critical locations

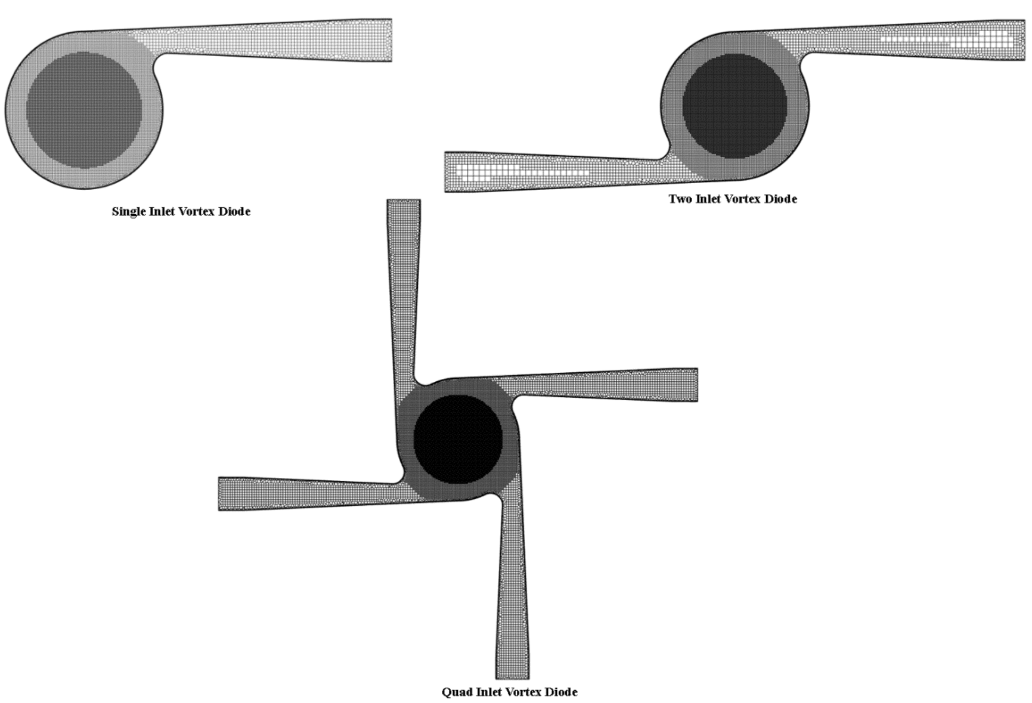

Figure S2(b). Mesh at the mid of the vortex chamber over horizontal plane for different number of inlets

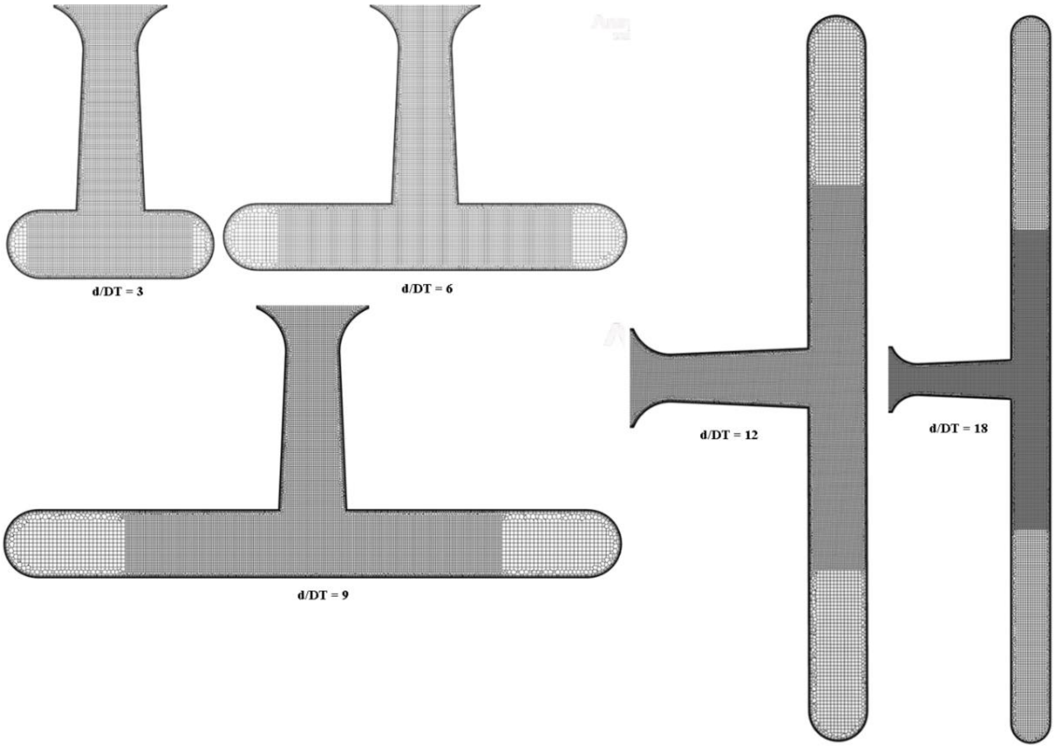

Figure S2(c). Mesh at the mid vertical plane for different D/d_T
